# Initiation of Long-Acting Antiretroviral Combinations Achieve Sustained Virologic Remission in Early-Life SIV Infection

**DOI:** 10.21203/rs.3.rs-9336128/v1

**Published:** 2026-04-14

**Authors:** Huanbin Xu, Xiaolei Wang, LI MA, Eunice Vincent, Yao-Zhong Liu, Lara Doyle-Meyers, Lori Rowe, Eddie Xu, Clara Krzykwa, Ronald Veazey, Mackenzie Cottrell, Jun Wang

**Affiliations:** Tulane University; Tulane National Primate Research Center; Tulane University; Tulane University School of Public Health and Tropical Medicine; Tulane National Primate Research Center; Tulane National Primate Research Center; Tulane University; Tulane University; Tulane National Primate Research Center; University of North Carolina at Chapel Hill; Rutgers, the State University

**Keywords:** HIV/SIV, infants, long-acting antiretrovirals, early treatment, viral remission

## Abstract

Long-acting (LA) antiretroviral agents offer a promising strategy to overcome adherence challenges in HIV prevention and treatment. However, their efficacy in early life has not been evaluated. Using a highly translational newborn macaque model of simian immunodeficiency virus (SIV) infection, we assessed the preventive and therapeutic potential of LA lenacapavir (LEN), alone or in combination with cabotegravir (CAB). Despite its efficacy in adult pre-exposure prophylaxis setting, early LEN monotherapy failed to prevent viral acquisition following intravenous SIV exposure at birth and resulted in virologic failure in infected infants. In contrast, combined administration of LA LEN and LA CAB rapidly suppressed viremia within 2 weeks and maintained undetectable viral loads for up to 1 month. Initiation of combination therapy at 2 days post infection led to rapid virus control and sustained virologic remission in majority of infants following analytical treatment interruption. By comparison, CAB-based regimens incorporating subtherapeutic doses of either LEN or rilpivirine (RPV) were ineffective in preventing infection. Together, these findings demonstrate that LA LEN monotherapy is insufficient for prevention or treatment following early-life HIV exposure and highlight the importance of early initiation of potent LA combination regimens to overcome adherence barriers and achieve potential pediatric HIV remission.

## INTRODUCTION

Despite prevention efforts, vertical transmission remains a persistent global challenge, with approximately 150,000 infants and children acquiring HIV annually, primarily during the intrapartum exposure (UNAIDS, 2021). Pediatric populations progress to AIDS-related illnesses substantially faster than adults if left untreated, suggesting the importance of early initiation of antiretroviral therapy (ART). However, daily ART regimens require frequent dosing and adherence, which are particularly challenging in early life. Long-acting (LA) therapies therefore represent a promising strategy to overcome these barriers.

LA antiretrovirals (ARVs), including cabotegravir (CAB), lenacapavir (LEN), and rilpivirine (RPV), have emerged as promising alternatives to daily therapy for HIV-1 pre-exposure prophylaxis (PrEP) and treatment^1, 2^. Clinical and nonhuman primate (NHP) studies have shown that single-dose LA CAB, LEN, or RPV can effectively reduce HIV/SIV acquisition when used as PrEP^3, 4, 5, 6, 7, 8, 9, 10, 11, 12, 13, 14, 15, 16, 17, 18, 19^. However, monotherapy with these agents is not recommended because of the risk of virologic failure and the emergence of drug resistance^5, 13, 20, 21^. Therefore, combination regimens are required to achieve durable viral suppression, particularly transitioning from daily ART^22, 23, 24, 25, 26, 27, 28, 29, 30, 31, 32, 33^. In support of early treatment strategies, we previously demonstrated that initiating a daily combined ART (cART, FTC/TDV/DTG) at 3 dpi resulted in ART-free viral remission in the majority of SIV-infected infant macaques^34, 35^. Nevertheless, the safety and efficacy of LA ARV regimens in developing infants remain largely undefined.

Pediatric NHP models provide a valuable translational platform for evaluating HIV prevention and treatment strategies, given their close genetic, physiological, and immunological similarities to humans (30–32). Utilizing a clinically relevant newborn macaque model, this study investigates early initiation of LA CAB/LEN and LA CAB/RPV combinations to suppress viral replication and evaluates their potential to achieve sustained virologic remission in a pediatric setting.

## RESULTS

### Predictions and structural analyses of LEN binding to the SIVmac251 capsid

Lenacapavir (LEN) binds to a highly conserved hydrophobic pocket located at the interface of two adjacent HIV-1 capsid (CA) subunits within the viral hexamer lattice. This pocket is formed by the N-terminal domain (NTD) of one CA monomer together with the NTD and C-terminal domain (CTD) of a neighboring monomer. Key residues mediating LEN interaction include Leu56 (L56), Asn57 (N57), Met66 (M66), Gln 67 (Q67), Lys 70 (K70), Asn 74 (N74), Thr107 (T107), and Asn 183 (N183)^17, 21, 36, 37^. To evaluate LEN binding to the SIVmac251 capsid, we performed structural modeling using the artificial intelligence–based prediction tool Chai-1^38^. The previously solved X-ray crystal structure of the apo SIV capsid protein (PDB: 7T14) revealed a hexameric architecture similar to that of the HIV-1 capsid^36, 39^. Using this structure as a template, we constructed a model comprising a hexameric capsid assembly with six CA monomers and six LEN molecules. The resulting model predicts that LEN binds within a pocket located at the interface between adjacent capsid subunits in the SIV hexamer (**Figs. 1A-1B**). Notably, the overall architecture of the predicted SIV capsid-LEN complex closely resembles that of the HIV-1 capsid bound to LEN (PDB: 6VKV), with nearly superimposable conformations of both the capsid proteins and the inhibitor^36^. Docking analysis indicates that LEN occupies the dimer interface of the SIV capsid in a binding pose highly similar to that observed in the HIV-1 capsid complex (**Fig. 1C**). LEN engages residues from both capsid monomers through a network of polar and hydrophobic interactions. Two hydrogen bonds are predicted between SIV Gag Asn191 (corresponding to CA N57) and the sulfone oxygen and amide NH groups of LEN, while the guanidinium NH of Arg204 (CA R70) forms a hydrogen bond with the amide carbonyl of the inhibitor (**Fig. 1D**). The 2,4-difluorophenylalaniline moiety of LEN is further stabilized by hydrophobic interactions with Leu190 (CA L56), Met200 (CA M66), and Ile207 (CA I73). Additional hydrophobic contacts from the adjacent monomer, including Tyr302 (CA Y168), Arg306 (CA R172), Ala312 (CA A178), and Lys315 (CA K181), interact with the bicyclic pyrazole ring of LEN. Together, these interactions support a high degree of structural conservation between the predicted SIV and known HIV-1 capsid-LEN complexes.

Given this structural conservation and the similarity of capsid sequences among SIV, SHIV, and HIV-1^17,18, 40^, we next evaluated the antiviral activity of LEN (GS-6027) against virulent SIVmac251 in primary rhesus PBMCs *in vitro*. As shown in **Supplementary Figure 1** and **Supplementary Table 2**, LEN exhibited potent antiviral activity with a mean 50% effective concentration (EC50) of 0.161 ng/mL. Dose-response analysis yielded a 95% effective concentration (EC95) of 2.032 ng/mL and a mean Hill slope of 1.4.

Considering plasma protein binding, the protein-adjusted LEN EC95 (paEC95) was estimated to be 19.71 ng/mL based on the 9.7-fold potency shift previously observed in rhesus and pig-tailed macaque models^18, 19^. This paEC95 is slightly higher than reported values for SHIV162p3 (8.8 ng/mL) and stHIV-A19 (1.46 ng/mL).

### Efficacy of a single dose of LEN, alone or combined with LA CAB, for prevention and treatment in infant macaques infected with SIV at birth

**Figure 2:**
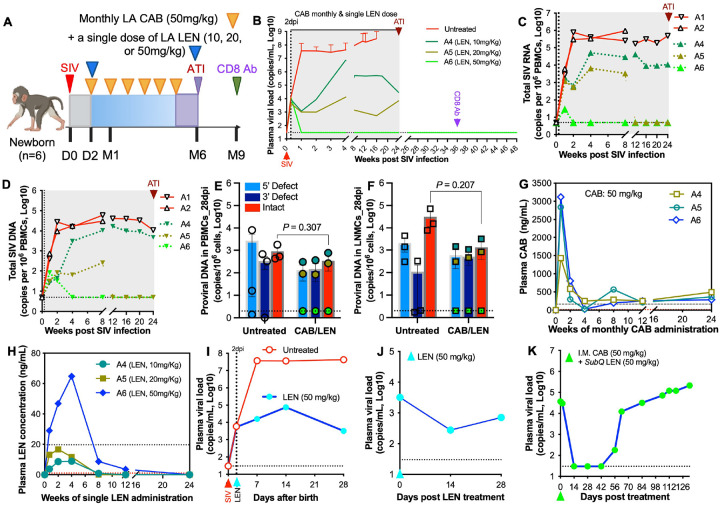
Efficacy of a single dose of LA LEN monotherapy or combined with LA CAB for prevention and treatment in SIV-inoculated newborn macaques. (**A**) Study design for preventive monthly LA CAB plus a single dose of LA LEN combination therapy in neonatal macaques for up to 6 months. Newborn macaques were intravenously inoculated with an identical SIVmac251 inoculum within 24 hours of birth. (**B**) Plasma viral load over a 6-month treatment course. The combination regimen (monthly intramuscular LA CAB, 50 mg/kg, combined with a single subcutaneous dose of LA LEN at 10, 20, or 50 mg/kg) was initiated at 2 dpi (*n*=3), and compared with age-matched untreated infant macaques (*n*=3). For infant A6, which exhibited viral remission after analytical treatment interruption (ATI), an anti-rhesus CD8α mAb (MT807R1) was administered intravenously by 12 weeks post-ATI, no viral blips or rebound were observed. Dotted line represents the assay LOD (30 copies per mL). (**C-D**) Dynamics of cell-associated (CA) SIV RNA (C) and SIV DNA (D) in PBMCs from individual animals (untreated, *n*=2; LA CAB/LEN, *n*=3). Dotted line represents the assay LOD (5 copies per million cells). (**E-F**) Levels of intact proviral DNA at 28 dpi in PBMCs (E) and lymph node-derived mononuclear cells (LNMCs) (F) (untreated, *n*=3; LA CAB/LEN, *n*=3). Dotted line represents the assay LOD (2 copies per million cells). (**G-H**) Plasma CAB (G) and LEN (H) concentrations over time in individual rhesus macaques receiving monthly LA CAB (50mg/kg) plus a single dose of LA LEN (10, 20, or 50 mg/kg), measured by mass spectrometry. The red dotted line represents the assay limit of detection (LOD: CAB, 12.5 ng/mL; LEN, 1 ng/mL). The upper dashed line represents defined therapeutic thresholds (CAB PA-IC90, 166 ng/mL, based on plasma levels observed in humans; rhesus LEN paEC95, 19.71 ng/mL). (**I-K**) Plasma viral load in individual infant macaques receiving a single 50 mg/kg dose of LA LEN: preventive monotherapy at 2 days in one neonate post SIV inoculation at birth, compared with untreated control (I); treatment monotherapy in one SIV-infected infant macaque (J); and treatment combined with CAB (50mg/kg) in one SIV-infected infant macaque (K). Dotted line represents the assay LOD (30 copies per mL). Statistical comparisons between treated and untreated groups at single time points (E-F) were performed using unpaired two-tailed Student’s t-tests. Longitudinal data (B-D, G-K) are presented descriptively due to limited sample size. Data are shown as mean ± SEM where applicable.

Given the risk of virologic failure associated with LA ARV monotherapy (*e.g*., CAB, LEN, or RPV) and the dosing regimens previously evaluated in adult NHP models^5, 13, 18, 19, 21, 41, 42^, we assessed the efficacy of early initiation of an LA CAB/LEN combination regimen for viral prevention and suppression in infant macaques. Newborn macaques were intravenously inoculated with SIV at birth and received monthly LA CAB (50 mg/kg) beginning at 2 dpi combined with a single dose of LA LEN at 10, 20, or 50 mg/kg for up to six months (Fig. 2A). The LA CAB-based regimen containing 50 mg/kg LA LEN rapidly suppressed viral replication, with plasma viral loads becoming undetectable by 7 dpi, PBMC-associated SIV RNA undetectable by week 2, and cellular SIV DNA undetectable by week 4 post infection. This animal subsequently achieved sustained viral remission following analytical treatment interruption (ATI), with no viral rebound detected even after *in vivo* CD8^+^ T cell depletion using an anti-rhesus CD8 antibody. In contrast, two animals receiving 10 mg/kg or 20 mg/kg LA LEN failed to achieve durable viral suppression during treatment, although cell-associated viral RNA and DNA in PBMCs became undetectable after approximately three months of therapy in animal receiving 20 mg/kg LA LEN (Figs. 2B–2D). These treatment outcomes were consistent with the detection of viremia as early as 7dpi and intact proviral DNA in systemic and lymphoid tissues by 28dpi (Figs. 2E–2F).

To characterize the pharmacokinetic (PK) profile of LA CAB/LEN administration, plasma concentrations of both drugs were measured and compared with reported human and adult macaque data. Plasma CAB concentrations showed similar PK profiles across the three infants, with rapid absorption peaking at approximately four times the protein-adjusted 90% inhibitory concentration (4×PA-IC90; 680 ng/mL, based on plasma levels observed in humans receiving 400mg monthly CAB) at week 1 following administration (50 mg/kg). This peak was followed by a rapid decline by week 2 and continued reduction to levels below the PA-IC90 by week 4, just prior to the next dosing cycle (Fig. 2G). For animals receiving a single 10 or 20 mg/kg dose of LEN, plasma concentrations peaked at approximately week 2 but remained largely below the protein-adjusted EC95 (paEC95; 19.71 ng/mL). In contrast, the 50 mg/kg dose produced peak plasma concentrations at approximately week 4 and maintained levels above the paEC95 from 1 to 7 weeks. However, LEN exposure in infants was shorter than that reported in adults, with plasma levels declining to largely undetectable levels by approximately 3 months post-administration (Fig. 2H).

Converging evidence indicates that a single subcutaneous LA LEN dose provides effective HIV/SHIV PrEP^7, 9, 17, 18, 19^ and therapeutic benefit in virologically suppressed patients^5, 21, 42, 43^. However, its preventive and therapeutic efficacy in pediatric settings remains unclear. Recent NHP studies demonstrated that a single 25 mg/kg LA LEN dose protects pig-tailed macaques from intravenous stHIV-A19 challenge^18^ and that 50–75 mg/kg doses achieve comparable PK in rhesus macaques^19^. Based on these findings, we evaluated a single 50 mg/kg LA LEN dose for early post-exposure prevention (PEP) and treatment in infant macaques. Administration of a single 50 mg/kg dose of LA LEN at 2 dpi in a neonate infected within 24 hours of birth failed to prevent infection (Fig. 2I). Similarly, LA LEN monotherapy did not achieve virus control when administered to a SIV-infected infant macaque, although transient reductions in plasma viral loads were observed (Fig. 2J). In contrast, combined administration of LA LEN and LA CAB rapidly reduced plasma viral loads to undetectable levels within two weeks and maintained aviremia for approximately one month (Fig. 2K). These findings indicate that an LA CAB/LEN combination regimen, rather than LA ARV monotherapy, may provide more durable antiviral activity in pediatric HIV infection.

### Early initiation monthly LA CAB combined with quarterly LA LEN in infant macaques exposed to and infected with SIV

**Figure 3:**
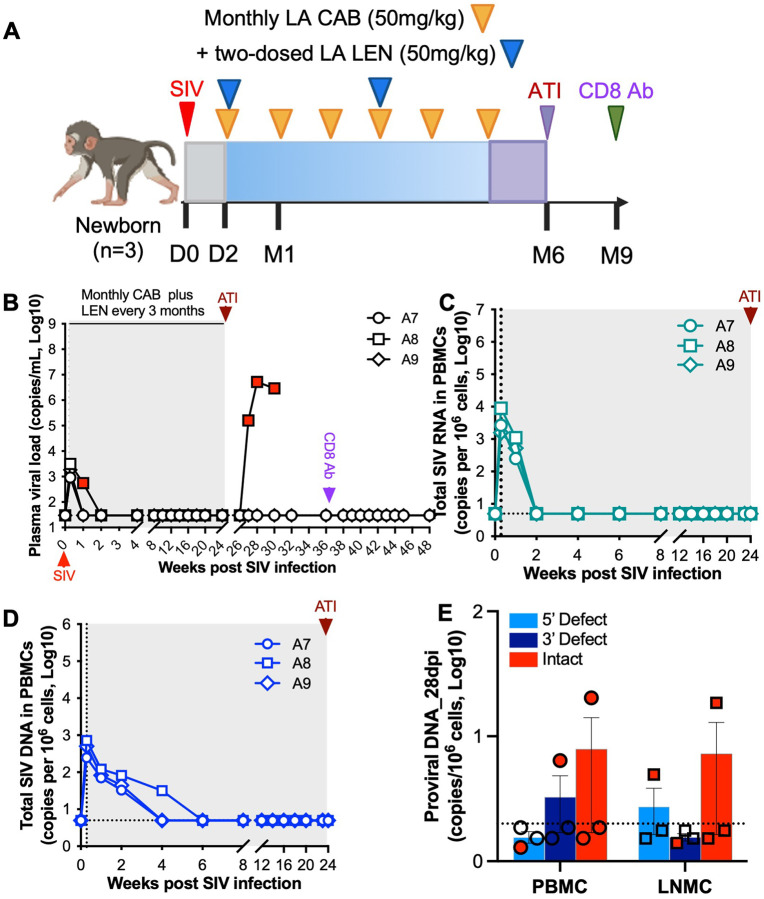
Efficacy of monthly LA CAB combined with quarterly LA LEN prevention intervention in SIV-inoculated newborn macaques. (**A**) Study design for preventive monthly LA CAB and quarterly LA LEN combination therapy in neonatal macaques for up to 6-month treatment course. Newborn macaques (*n*=3) were intravenously inoculated with an identical SIVmac251 inoculum within 24 hours of birth. A combination regimen, consisting of monthly intramuscular LA CAB (50 mg/kg) and a single subcutaneous dose of LEN (50 mg/kg) every 3 months, was initiated at 2 dpi and continued for up to 6 months. (**B**) Plasma viral load in individual infant macaques over the 6-month treatment course. One infant, A8, experienced viral rebound after ATI. Two other virus-controlled infant macaques received anti-rhesus CD8α mAb intravenously by 12 weeks after ATI, and no viral blips or rebound were observed. Dotted line represents the assay LOD (30 copies per mL). (**C-D**) Dynamics of cell-associated total SIV RNA (C) and SIV DNA (D) in PBMCs of individual animals. Dotted line represents the assay LOD (5 copies per million cells). (**E**) Levels of intact proviral DNA at 28 dpi in PBMCs and LNMCs. Dotted line represents the assay LOD (2 copies per million cells). Due to the small sample size, all data are presented descriptively without formal statistical testing. Individual animal trajectories are shown.

Because the antiviral effect of a single 50 mg/kg dose of LA LEN persisted for only ~3 months in infant macaques, we repeated the early initiation study using the same LA CAB/LEN regimen but modified the dosing schedule to administer 50 mg/kg LA LEN every three months during a six-month treatment course (Fig. 3A). Among the three treated infants, one animal exhibited detectable plasma viremia from 7 to 14 dpi before achieving aviremia. This delayed viral suppression was subsequently associated with viral rebound following treatment interruption. In contrast, the other two LA CAB/LEN-treated infants achieved rapid viral suppression to undetectable levels by 7 dpi and maintained aviremia with no viral rebound, even after in vivo CD8^+^ cell depletion (Fig. 3B). In these two infant animals, aviremia at 7 dpi was accompanied by rapid decline of PBMC-associated SIV RNA by 14 dpi and undetectable intact proviral DNA in both PBMCs and LNMCs by week 4. In contrast, one animal, experiencing viral rebound after ATI, showed delayed viral suppression until 14 dpi, with persistent total SIV DNA and intact proviral DNA detectable at week 4 (Figs. 3C–3E). Collectively, these findings suggest that efficacy of early LA CAB/LEN initiation correlates with early virologic control and rapid decay of early viral reservoirs in peripheral and lymphoid tissues, resulting in sustained virologic remission in infant macaques exposed to and infected with SIV at birth. However, further optimization of LA CAB and LA LEN dosing regimens will be required to maximize efficacy and reduce the risk of virus acquisition and viral rebound in clinically relevant pediatric settings.

### Testing the efficacy of an alternative LA CAB/RPV regimen in infant macaques infected with RT-SHIV at birth

**Figure 4:**
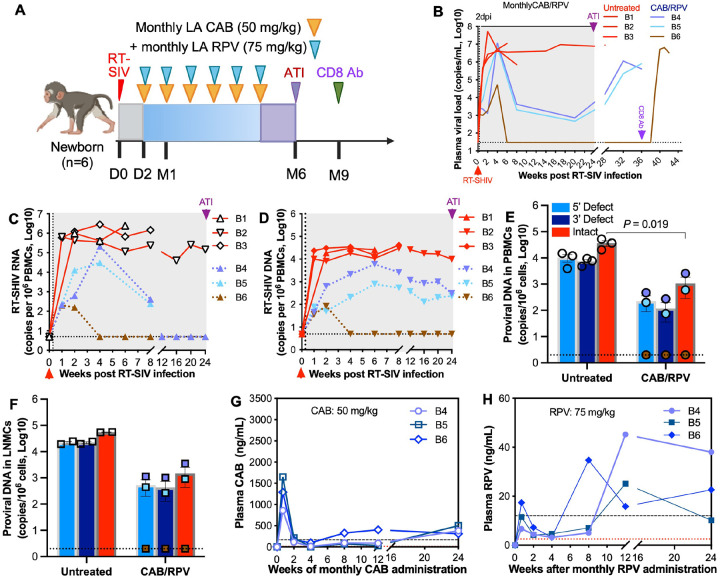
Efficacy of monthly LA CAB combined with monthly LA RPV prevention intervention in RT-SHIV inoculated newborn macaques. (**A**) Study design for preventive monthly LA CAB and monthly LA RPV combination therapy in neonatal macaques for up to 6-month treatment course. Newborn macaques were inoculated with 200 TCID50 RT-SHIV. One group of neonatal macaques (*n*=3) received a combination regimen consisting of monthly intramuscular LA CAB (50 mg/kg) and monthly intramuscular LA RPV (75 mg/kg), starting at 2 dpi and continuing for up to 6 months. This group was compared with age-matched, untreated infant macaques (*n*=3). (**B**) Plasma viral load over the 6-month treatment course. Only one infant, B6, showed viral remission after ATI. However, viral rebound was detected at week 3 following anti-rhesus CD8α mAb administration. Dotted line represents the assay LOD (30 copies per mL). (**C-D**) Dynamics of cell-associated total RT-SIHV RNA (C) and RT-SIHV DNA (D) in PBMCs of individual animals (untreated, *n*=3; LA CAB/RPV, *n*=3). Dotted line represents the assay LOD (5 copies per million cells). (**E-F**) Levels of intact proviral DNA at 28 dpi in PBMCs (E) and LNMCs (F) isolated from untreated or LA CAB/RPV-treated animals. Dotted line represents the assay LOD (2 copies per million cells). (**G-H**) Plasma LA drug concentrations over time for CAB (G) and RPV (H) in individual infant macaques receiving monthly CAB and monthly RPV. The bottom red dotted line indicates the assay limit of detection (LOD: CAB, 12.5 ng/mL; RPV, 2.5 ng/mL). The upper dashed line denotes the predefined therapeutic thresholds (PA-IC90: CAB, 166 ng/mL; RPV, 12 ng/mL). Statistical comparisons between treated and untreated groups at single time points (E and F) were performed using unpaired two-tailed Student’s t-tests. Longitudinal virologic and pharmacokinetic data (B-D, G-H) are presented descriptively due to limited sample size. Data are shown as mean ± SEM where applicable.

Compared with LA CAB/LEN combinations, the LA CAB/RPV regimen, consisting of an integrase strand transfer inhibitor and a non-nucleoside reverse transcriptase inhibitor, is widely used for HIV treatment in virologically suppressed patients^26, 29, 32, 44, 45, 46, 47^. Given the efficacy of CAB-based combinations appears to depend on the companion drug dose and early treatment initiation (*e.g*., 2 dpi), we evaluated whether a LA CAB/RPV regimen could achieve viral suppression in infant macaques infected with RT-SHIV at birth. Previous studies showed that a single 50 mg/kg dose of LA RPV failed to achieve plasma concentrations above 12 ng/mL (the equivalent PA-IC90 in humans)^48^, whereas higher doses of LA RPV (100–200 mg/kg) were effective for both PrEP and treatment in adult macaque RT-SHIV models^13, 30, 33^. Based on these observations and the minimal dosing volume feasible in infant animals, we tested a regimen consisting of monthly intramuscular administration of 50 mg/kg LA CAB combined with 75 mg/kg LA RPV in infant macaques for up to six months (Fig. 4A). This LA CAB/RPV combination failed to fully suppress viral replication in two of the three infant macaques throughout the treatment. The third animal achieved undetectable plasma viral loads by approximately 6 weeks (~42 days) after treatment initiation and maintained aviremia through the six-month treatment course. However, viral rebound occurred three weeks after *in vivo* CD8^+^ cell depletion (Fig. 4B). Notably, early virologic markers remained detectable in virologically suppressed animal, including plasma viremia at 7dpi, and CA SIV RNA at 14 dpi (Figs. 4C–4D). Although intact proviral DNA became undetectable in PBMCs and LNMCs by approximately week 4 after treatment initiation (Figs. 4E–4F), subsequent viral rebound suggests that early viral reservoirs were established in other tissues, such as tonsils^34^, likely due to incomplete suppression of viral replication during the initial days of infection. These early virologic indicators were consistent with treatment outcome predictors observed in the LA CAB/LEN cohorts (Figs. 1 and 2).

PK analysis showed that plasma CAB profiles in the LA CAB/RPV-treated infants were comparable to those observed in the LA CAB/LEN-treated cohorts (Fig. 4G). However, during the first four weeks after the 75 mg/kg dose, plasma RPV concentrations remained largely below the PA-IC90 observed in humans receiving monthly 400 mg RPV or in adult macaques given 100 mg/kg LA RPV. The only exception was one infant animal at day 5, which transiently exceeded this threshold and showed relatively improved virologic suppression. LA RPV PK profiles also displayed substantial inter-individual variability among infant macaques (Fig. 4H). Consistent with these observations, RPV exposure during the first month highly correlated with viral reservoir levels in PBMCs and LNMCs. Collectively, these findings indicate that early initiation of LA CAB/RPV regimen with a 75 mg/kg RPV dose results in subtherapeutic plasma RPV concentrations, leading to incomplete viral suppression, persistence of viral reservoirs, and viral rebound following CD8^+^ cell depletion.

## DISCUSSION

Long-acting (LA) antiretroviral therapies provide a strategic advantage for HIV prevention and treatment by overcoming the adherence challenges associated with frequent dosing. This preclinical study addresses a central challenge in pediatric HIV prevention and treatment: whether LA ARV strategies, initiated in the early stage of life, can achieve rapid viral suppression and enable sustained virologic remission. Specifically, we asked: 1) whether LA LEN monotherapy is sufficient for early prevention or treatment of infection; 2) whether combination LA therapy with CAB and LEN can substantially reduce HIV-1 acquisition risk and improve virologic outcomes, and 3) how regimen composition, including alternative LA RPV dosing, affects antiviral efficacy in a highly translational newborn macaque model of early-life SIV infection.

Our previous study established a newborn macaque model in which animals are intravenously inoculated with SIV within 24 hours of birth, resulting in detectable viremia and cell-associated viral DNA/RNA as early as 1 dpi^34^. Using this highly translational model, our findings provide several key insights. First, LA LEN monotherapy was insufficient to prevent infection or achieve durable virus control in infected infants, reinforcing the limitations of single agent LA regimens for both prevention and treatment. Second, combination therapy with LA CAB and LA LEN exhibited potent antiviral activity, rapidly reducing plasma viremia to undetectable levels within two weeks and maintaining viral suppression for approximately one month in infected infants following a single-dose administration. Notably, when initiated as early as 2 days post infection, this regimen only achieved rapid control of viral replication but also enabled sustained treatment-free remission in the majority of treated infant macaques. In contrast, CAB-based regimens incorporating subtherapeutic dosing of LA LEN or LA RPV demonstrated limited efficacy, highlighting the importance of achieving adequate drug exposure and potency in LA combination strategies. Taken together, these findings support the potential of early initiation of LA CAB/LEN combination therapy as both preventive and therapeutic approach for pediatric populations exposed to or infected with HIV.

The HIV/SIV capsid (CA) protein plays a critical role in multiple stages of the viral life cycle and is essential for viral infectivity^49, 50, 51^. LEN inhibits viral replication by binding to a conserved hydrophobic pocket at the interface of adjacent CA monomers, thereby disrupting CA-mediated interactions required for viral assembly and replication^21, 36^. Structural studies have identified direct LEN contacts with residues N57, M66, Q67, K70, and N74, while neighboring residues, including L56, A105, and T107, as well as residues from adjacent monomers (S41, Q179, N183) stabilize the binding interface^21, 36, 37, 52, 53^ Resistance-associated substitutions such as L56I, M66I, Q67H, K70N, N74D/S, and T107N have been reported^54, 55^. Although most of these residues are highly conserved between HIV-1 and SIV, residue 70 differs (HIV-1 K70 versus SIV R70)^17, 18^. Structural modeling suggests that SIV R70 can still participate in LEN binding. Consistent with this prediction, GS-6027 (LEN) exhibited potent antiviral activity against SIVmac251, although slightly reduced compared with reported potency against SHIV162p3 and simian-tropic HIV^17, 18, 19^. In this study, capsid sequencing of plasma virus from LEN-treated infant macaques experiencing virologic failure revealed no treatment-emergent resistance mutations (data not shown), suggesting that virologic failure likely resulted from insufficient antiviral exposure or suboptimal drug concentration rather than resistance selection.

While LA antiretroviral agents demonstrate strong efficacy for HIV/SIV PrEP^8, 9, 13, 15, 17, 19, 56, 57^, treatment with LA CAB, LEN, or RPV alone typically reduces but does not fully suppress viral replication^5,13, 21, 41, 42^. Combination therapy is therefore required to achieve durable virus control and limit resistance development in clinical settings^31, 32, 42, 47, 58, 59, 60^. Consistent with this principle, a single subcutaneous dose of LA LEN in an SIV-infected infant macaque resulted in only an approximately one-log reduction in plasma viral load, whereas the LA CAB/LEN combination rapidly suppressed viral replication to undetectable levels. Similarly, LA LEN administered alone at 2 dpi failed to prevent infection following early viral exposure. Considering integrated proviral DNA serves as the template for persistent HIV replication and becomes extremely difficult to eliminate once established^61^, combining a capsid inhibitor (LEN) with an integrase strand transfer inhibitor (CAB) provides a mechanistically complementary strategy that simultaneously blocks viral genome integration and viral replication.

Despite the promise of LA regimens, limited data exist to guide their use for early prevention or treatment in neonates at high risk of vertical HIV transmission. In this study, monthly administration of LA CAB at 50 mg/kg produced relatively consistent PK profiles across treatment cohorts, regardless of the companion drug. However, reduced LA LEN doses (10–20 mg/kg) in CAB-based regimens resulted in lower peak plasma concentrations and shorter drug exposure, which were associated with virologic failure, even when treatment was initiated early. Similarly, a LA CAB-based regimen containing a subtherapeutic 75 mg/kg LA RPV dose failed to achieve durable virus control, in contrast to effective RPV doses of 100–200 mg/kg reported in adult macaques^13, 30, 33^. Viral rebound following treatment discontinuation or *in vivo* CD8^+^ cell depletion suggests that insufficient drug exposure permitted establishment of persistent viral reservoirs. Additionally, a regimen consisting of monthly CAB combined with LA LEN (50 mg/kg each) administered every three months achieved durable virus control and treatment-free remission in three of four infant macaques. One animal experienced viral rebound following ATI, likely reflecting individual variability in LA LEN exposure during the initial days of administration, although pharmacokinetic measurements were unavailable for this animal.

Pharmacokinetic analyses revealed that detectable plasma LEN levels persisted for approximately three months in infant macaques, shorter than the ~ six-month duration reported in adults receiving comparable doses^19^. These differences may reflect developmental variations in drug absorption, distribution, metabolism, and excretion (ADME)^62^. For example, neonates may exhibit altered activity of hepatic enzymes such as CYP3A4 (Cytochrome P450 3A4 enzyme) and UGT1A1 (Uridine diphosphate glucuronosyltransferase 1A1 enzyme) that contribute to LEN metabolism^59, 63, 64, 65, 66^. Notably, early viremia at 7 dpi strongly correlated with plasma concentrations of LA ARVs and treatment outcomes. In addition, LEN PK, including peak and duration, were closely associated with dose. All of these data suggest that early drug exposure, together with viremia status within the first week of treatment, may predict long-term treatment outcomes, including the likelihood of sustained virologic remission. Given the slow-release pharmacokinetics and dose-proportional exposure of subcutaneous LA LEN^67, 68^, these findings suggest that higher initial LA LEN dosing (*e.g*., 75–100 mg/kg), at least at treatment initiation, may be necessary to ensure more effective viral suppression in HIV-exposed infants.

Together with our previous studies demonstrating sustained virologic remission following early daily ART in this pediatric nonhuman primate model, the present work provides proof-of-concept that early initiation of potent LA combination antiretroviral therapy can fundamentally alter the outcomes of perinatal HIV infection. It also validates the newborn macaque model as a powerful and clinically relevant platform for evaluating pediatric HIV cure and remission strategies. Importantly, the data suggest that early-life therapeutic intervention with optimized LA combinations may reduce viral reservoir establishment and increase the likelihood of viral remission. Despite these advances, several limitations should be considered. The sample size inherent to nonhuman primate studies was limited, which may constrain the generalizability of the findings. The follow-up period, while sufficient to demonstrate 6-month of viral remission thus far, does not fully address the durability of long-term virus control or the potential for late viral rebound. In addition, while pharmacokinetic and virologic outcomes were carefully assessed, deeper characterization of viral reservoirs, immune responses, and tissue-specific drug distribution remains incomplete. Finally, although the newborn macaque model closely recapitulates key aspects of pediatric HIV infection, species-specific differences must be considered when translating these findings to human infants. Future research should build on these findings by optimizing dosing strategies and formulations of LA CAB/LEN to extend the duration of viral suppression and improve therapeutic coverage. Larger and longer-term studies are needed to evaluate the durability of treatment-free remission and to identify predictors of post-treatment control. In addition, exploration of multi-drug LA combinations, including next-generation agents, may further enhance efficacy. Ultimately, clinical translation will require carefully designed trials to assess the safety, pharmacokinetics, and efficacy of early LA antiretroviral interventions in neonates at risk of or living with HIV. In summary, while LEN has recently been approved for HIV prevention in adolescents at risk of infection^69^, this study demonstrates that LA LEN monotherapy is inadequate, early initiation of a potent LA CAB/LEN combination can achieve rapid viral suppression and promote sustained treatment-free remission in a neonatal model of SIV infection. These findings provide a strong rationale for advancing early-life LA combination strategies as a transformative approach toward durable HIV remission in pediatric populations.

## MATERIALS AND METHODS

### Ethics Statement

All animals in this study were housed at the Tulane National Biomedical Research Center in accordance with the Association for Assessment and Accreditation of Laboratory Animal Care International standards. All studies were reviewed and approved by the Tulane University Institutional Animal Care and Use Committee under an approved protocol (P0524). Animal housing and studies were carried out in strict accordance with the recommendations in the Guide for the Care and Use of Laboratory Animals of the National Institutes of Health (NIH, AAALAC #000594). All clinical procedures were carried out under the direction of a laboratory animal veterinarian. All procedures were performed under anesthesia using ketamine, with or without dexmedetomidine hydrochloride, or tiletamine hydrochloride, and analgesics (buprenorphine) were administered for all potentially painful procedures. All efforts were made to minimize stress, improve housing conditions, and to provide enrichment opportunities (e.g., objects to manipulate in cages, varied food supplements, foraging and task-oriented feeding methods, interaction with caregivers and research staff).

### Viruses and drugs

Virulent SIVmac251 stocks were obtained from TNBRC core. CCR5-tropic reverse transcriptase simian-human immunodeficiency virus (RT-SHIV), containing the reverse transcriptase (RT) of HIV-1 HXB2 that is sensitive to RPV, was obtained through the National Institutes of Health AIDS Reagent Program, Division of AIDS, NIAID, NIH (Catalog No. ARP-11342), contributed by Dr. Thomas North and Dr. Joseph Sodroski. CAB LA (200 mg/mL), LEN LA (309 mg/mL), and RPV LA (300 mg/mL) were purchased from Cardinal Specialty Health/Tulane Pharmacies as single-dose kit.

### Modeling of SIVmac251 gag and lenacapavir interactions

The SIV capsid protein sequence used for modeling is retrieved from UniProt under the ID P05893. The complex structure of the SIV capsid protein with lenacapavir was modeled using CHAI^38^, employing six copies of the capsid protein and six copies of the ligand. The overlay structures were generated using Pymol.

### Anti-SIVmac251 antiviral assay in rhesus peripheral blood mononuclear cells *in vitro*

Rhesus PBMCs were isolated from rhesus macaques (n=3) intrarectally infected with SIVmac251 at day 7 post infection, with detectable plasma viral load of 4.8eE+02, 3.6e+03, 4.2E+02 copies/mL. The PBMCs were suspended with complete RPMI and seeded into 48-well Multiple Well plates (Not No: 3388, Corning) at a cell density of 5 × 10^5^ cells per well in 500 μL. Ten-point threefold serial dilutions of GS-6027 compound (Cat. No. HY-111962, Medchemexpress) were added in triplicate to wells of cell culture medium containing human recombinant 10 ng/mL IL-2 (Cat No: 78145, STEMCELL). The cell cultures were incubated in a 5% CO_2_ incubator at 37 °C for 7 days. The cell-free supernatants were harvested 7 days after culture, the SIV RNA in culture supernatants was quantified using digital PCR. The mean EC50 for GS-6027 was determined by the dose–response curves using a 4-parameter curve fit in GraphPad Prism 10.3 software. The slope of the curve for the Hill coefficient (*n*) (n = 1.4 ± 0.156) was used to calculate the EC_95_ value by equation of the EC95 = [95/(100–5)^1/*n*^ × EC_50_ and the protein-adjusted EC95 (paEC95) based on the 9.7-fold reduced potency of LEN in macaque models *in vivo*^18, 19^.

### Study design

A total of 19 Indian-origin infant rhesus macaques (*Macaca mulatta*) were included in this study (Supplementary Table 1). Newborn macaques were intravenously inoculated within 24 hours of birth with 100 TCID50 SIVmac251 (n=12) or 200 TCID50 RT-SHIV (n=6). An SIV-naïve 6-month-old infant animal received a single 50 mg/kg dose of both LA CAB and LA LEN for PK evaluation. The virus-inoculated newborns infant animals include age-matched untreated controls following infection with SIVmac251 (n=3) or RT-SHIV (n=3). Among the SIVmac251-inoculated newborns, three animals initiated a six-month LA combination therapy beginning at 2 dpi, consisting of monthly LA CAB (50mg/kg, intramuscularly intramuscular injection alternating between the right and left thigh) and a single dose of LA LEN (10, 20, or 50 mg/kg, subcutaneously/*SubQ* injection on the dorsum). A second SIVmac251-infected neonatal cohort (n=3) received monthly LA CAB (50 mg/kg) together with two doses of LA LEN (50 mg/kg, administered every three months) during the six-month treatment course. For prevention and treatment pilot studies, a single 50 mg/kg dose of LA LEN was administered: to a neonatal macaque exposed to SIV at birth (n=1), to an SIV-infected infant macaque (n=1), and to an SIV-infected infant macaque (n=1) in combination with a single 50 mg/kg dose of LA CAB. For comparison, RT-SHIV infected neonatal macaques (n=3) received a regimen of monthly LA CAB (50 mg/kg) and monthly LA RPV (75 mg/kg), both administered intramuscularly, starting at 2 dpi and continuing for up to six months. Blood and axillary lymph node samples were collected weekly or biweekly.

### *In vivo* CD8+ lymphocyte depletion

To deplete CD8+ lymphocytes (CD8+ T cells and NK cells), rhesus macaques were administered by a single intravenous 50 mg/kg dose of anti-rhesus IgG1 CD8α monoclonal antibody (MT807R1, Cat No: PR-0817, NIH NHP Reagent Resource)^34^. Absolute CD8+ T-cell counts (Ab clone, SK1 and RPA-T8), and viral loads were measured following MT807R1 infusion.

### Nucleic acid extraction of plasma and cell samples

Peripheral mononuclear cells (PBMCs) and lymph node-derived mononuclear cells (LNMCs) were processed for extraction of genomic DNA and total RNA using the AllPrep DNA/RNA Mini Kit (Cat No: 80311, Qiagen). In brief, cell samples were homogenized in 700 μL Buffer RLT Plus supplemented with 80 μL Proteinase K solution (20 mg/mL, Invitrogen, Cat. No. 2935092), followed by DNA and RNA isolation according to the manufacturer’s instructions. Viral RNA from plasma was extracted using the QIAamp Viral RNA Mini Kit (Qiagen, Cat. No. 52962). All DNA and RNA samples were stored at −80 °C until use.

### Detection of plasma viral load and cell-associated SIV DNA/RNA

Specific primer and probe sets targeting the SIV *gag* region^34, 35^ and rhesus RPPR30 were synthesized by integrated DNA technologies. The sequences were as following: SIV gag: Forward, GTC TGC GTC ATC TGG TGC ATT C; Reverse, CAC TAG GTG TCT CTG CAC TAT CTG TTT TG; and probe, FAM-CTT CCT CAG TGT GTT TCA CTT TCT CTT CTG CG-BHQ-1. Rhesus RPPR30: Forward, TCA GCA TGG CGG TGT TT; Reverse, GCT GTC TCC ACA AGT C; and probe, VIC-TTC TGA CCT GAA GGC TCT GCG C-3IABkFQ-1. The SIV gag primer/probe set was used to determine viral load in plasma (LOD, 30 copies/mL). Plasma viral RNA was reverse transcribed into cDNA using the ThermoFisher SuperScript^™^ VILO^™^ Master Mix (Invitrogen, Grand Island, NY). Reactions were performed on a thermocycler at 25 °C for 10 min, a 42 °C for 60 min, and enzyme inactivation at 85 °C for 5 min. cDNA or cellular DNA was used to quantify copies of SIV gag by digital PCR on the QuantStudio Absolute Q Digital PCR System (Applied Biosystems, Thermo Fisher, US) using the Absolute Q^™^ MAP16 Plate Kit and Master Mix. Cycling conditions were: 96 °C for 10 min, followed by 40 cycles of 96 °C for 15s and 60 °C for 30s. For cell samples, cellular input was normalized by quantifying RPPR30 genomic copies (two copies per rhesus macaque cell). Quantification of SIV DNA and SIV RNA was performed using a minimal of 2´10^5^ cell number equivalents per sample, and results were expressed as copies per one million cells (LOD, 5 copies/mL). The samples with undetectable or very low levels of SIV close to LOD, nested quantitative digital PCR, capable of detecting single-copy target, were further performed to confirm absence or accurately quantify low-level copies. To assess the emergence of SIV capsid gene mutations, plasma viral RNA from LEN-treated infant animals exhibiting viral rebound or persistent viremia was amplified using primers targeting the SIV capsid gene (forward: GAT AGT GCA GAG ACA CCT AG; reverse: TGG GGA AAT TGC GGG GCT TC). The resulting amplicons were sequenced to identify CA gene/protein mutations compared to those in untreated controls.

### Intact proviral DNA assay (IPDA)

The SIV-associated IPDA (SIV-IPDA) was used to determine the copies of intact SIV proviruses as described^70^. Genomic DNA in PBMCs and LNMCs with a minimal of 5´10^5^ cell number equivalents per sample was extracted using a QIAamp DNA Mini kit (Qiagen). DNA quantity was primarily evaluated by spectrophotometry. In brief, IPDA includes quantification of two conserved while frequently deleted regions of the SIV genome (copies of the intact provirus), levels of a 2-long terminal repeat (2-LTR) circular DNA, as well as input cell numbers and DNA shearing index referenced by ribonuclease P/MRP subunit P30 (RPP30). The intact proviral frequencies were expressed per million cells, based input cell counts, DNA shearing index and quantity of 2-LTR circles. Multiplex digital PCR (dPCR) reaction were performed using the QuantStudio Absolute Q Digital PCR System (Applied Biosystems, Thermo Fisher, US).

### Measurements of plasma LA drug concentrations

To monitor pharmacokinetic of long-acting antiretrovirals (ARVs) in infant macaques receiving CAB/LEN or CAB/RPV formulations, plasma CAB, LEN, and RPV concentrations were measured using chromatography-tandem mass spectrometry methods as previously described^71^. Free and protein-bound CAB, LEN and RPV will be extracted from 100 μL plasma samples by protein precipitation with isotopically labeled internal standards, separated using reverse-phase chromatography, and detected on an AB Sciex API-5000 triple quadrupole mass spectrometer using electrospray ionization in positive ion mode. Acceptance criteria for calibration standards and quality control samples will be set within 15% of nominal concentrations and 20% at the lower limit of quantification (LLOQ). PK analysis will be performed using WinNonlin software (Certara, Cary NC) to estimate absorption and clearance rates. Dynamic range of assay: 12.5–25,000ng/mL (CAB), 2.5–5,000ng/mL (RPV), 1–1,000ng/mL (LEN).

### Statistical analysis

Statistical analyses were performed using GraphPad Prism version 10.3 (GraphPad Software, San Diego, CA). All tests were two-sided, and a p-value <0.05 was considered statistically significant. Data are presented as mean ± standard error of the mean (SEM). Given the small sample sizes inherent to nonhuman primate studies, statistical analyses were primarily descriptive. For comparisons between treated and untreated groups at single time points (e.g., cell-associated viral RNA/DNA and intact proviral DNA levels), unpaired two-tailed Student’s t-tests were applied when appropriate. Longitudinal data, including plasma viral load and pharmacokinetic profiles, were analyzed descriptively without formal repeated measures modeling due to limited sample size. Dose–response curves were fitted using four-parameter logistic (Hill) model implemented in GraphPad Prism, and EC50 and EC95 values were estimated accordingly. No adjustments for multiple comparisons were applied, and results should be interpreted as exploratory. Given the limited sample size, statistical testing was not used to support conclusions from longitudinal outcomes, which were instead interpreted based on consistent biological trends across animals.

## Supplementary Material

Supplementary Files

This is a list of supplementary files associated with this preprint. Click to download.

- SupplTable1.jpg
- SupplTable2.jpg
- SupplFigure1.jpg

## Data Availability

All data are available in the main text or the supplementary materials.

## ABBREVIATIONS

SIV: simian immunodeficient virus
ARVs: antiretrovirals
CAB: cabotegravir
LEN: lenacapavir
RPV: rilpivirine
